# Supplementary figures and images for: Intracellular Staphylococcus aureus employs the cysteine protease staphopain A to induce host cell death in epithelial cells
Source: PLoS Pathog. 2021 Sep 2;17(9):e1009874. doi: 10.1371/journal.ppat.1009874 (PMC8443034; doi:10.1371/journal.ppat.1009874)

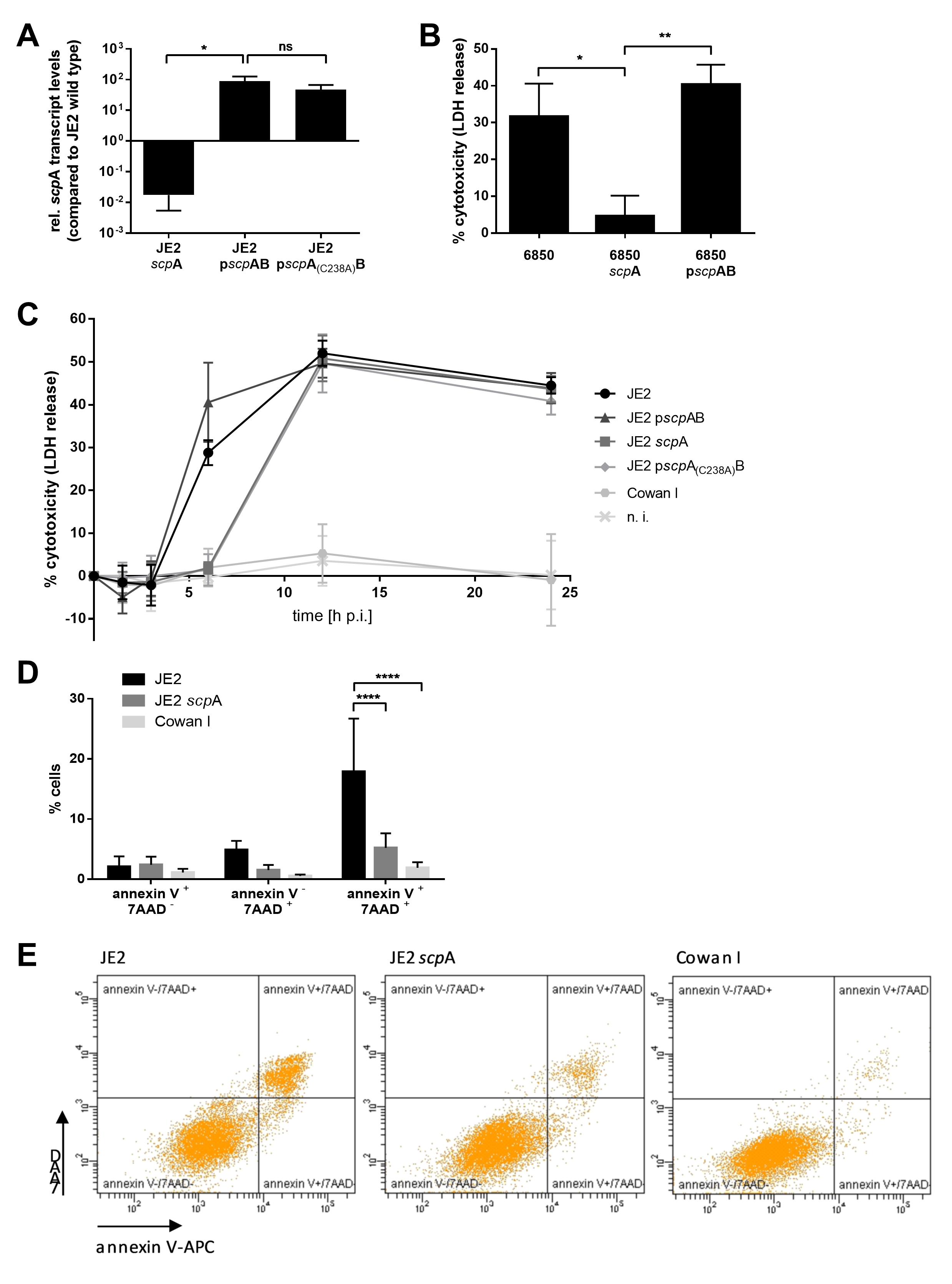

Supplement: S1 Fig — (A) RNA was isolated from bacterial overnight cultures and expression of scpA in JE2 scpA, JE2 phld-scpAB and JE2 phld-scpA(C238A)B was determined by qRT-PCR and normalized to scpA expression of JE2 wild type. (B) HeLa cells were infected with S. aureus 6850 wild type, transposon mutant of staphopain A (6850 scpA) or complemented mutant (6850 pscpAB) and cytotoxicity was determined by LDH release 6 h p.i. (C) HeLa cells were infected with wild type strain (JE2), staphopain A mutant (JE2 scpA), complemented mutant (JE2 pscpAB), complemented mutant with active site mutation (JE2 scpA(C238A)B) or Cowan I and cell death was assessed at 1.5, 3, 6, 12 and 24 h p.i. by LDH assay. (D, E) HeLa cells were infected with wild type (JE2), staphopain A mutant (JE2 scpA) or Cowan I and apoptotic cells were determined by annexin V-APC and 7AAD staining and flow cytometric analysis 6 h p.i. Statistical significance was determined by one-way ANOVA (A, B) or two-way ANOVA (D) (*P<0.05, **P<0.01, ****P<0.0001). (TIF) [file ppat.1009874.s001.tif]

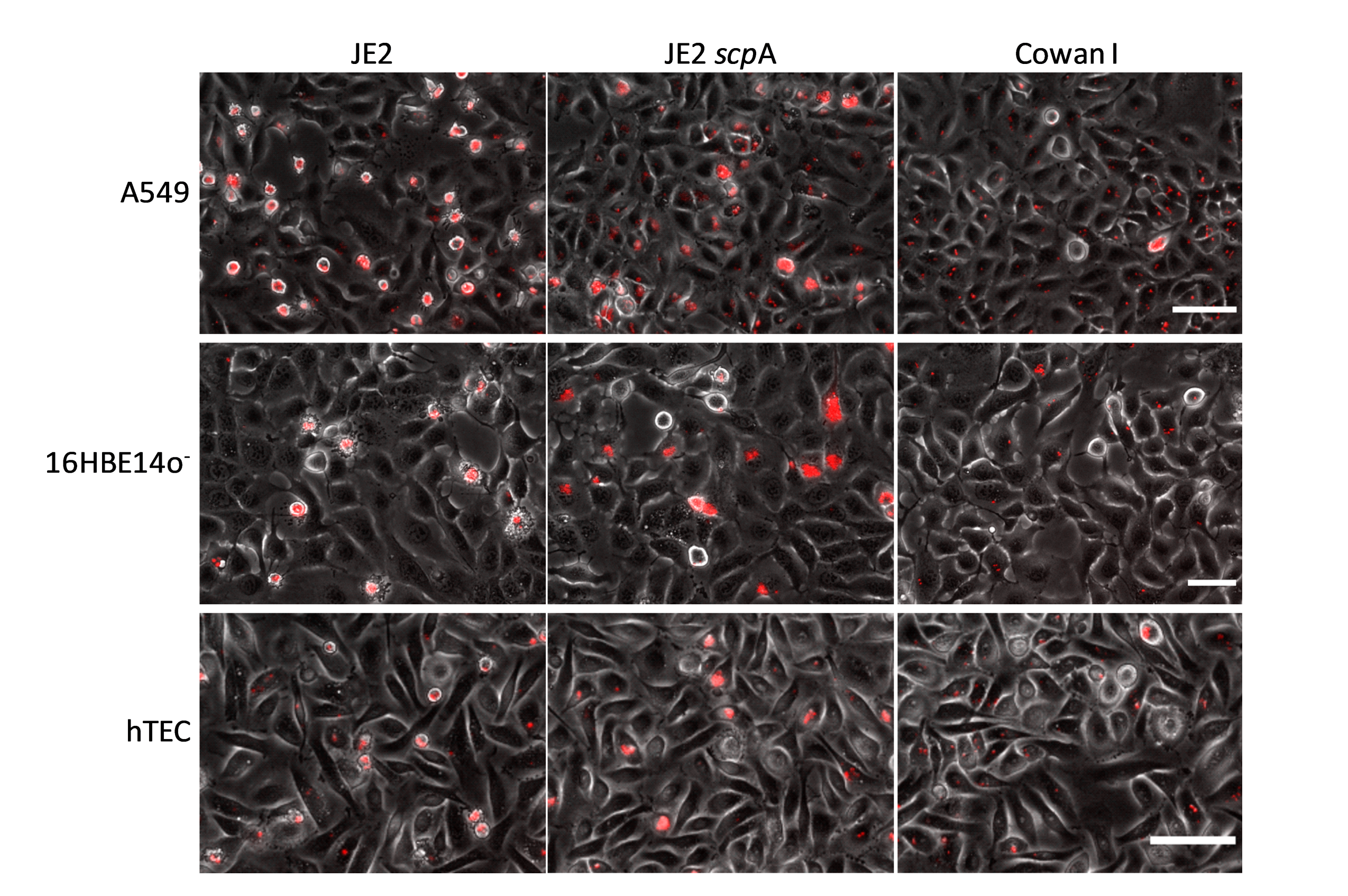

Supplement: S2 Fig — Microscopic images of A549, 16HBE14o- and hTEC cells infected with JE2 wild type, JE2 scpA or Cowan I expressing mRFP at 6 h p.i. (A549, 16HBE14o-) or 8 h p.i. (hTEC) (gray: PC, red: S. aureus, scale bar: 20 μm). (TIF) [file ppat.1009874.s002.tif]

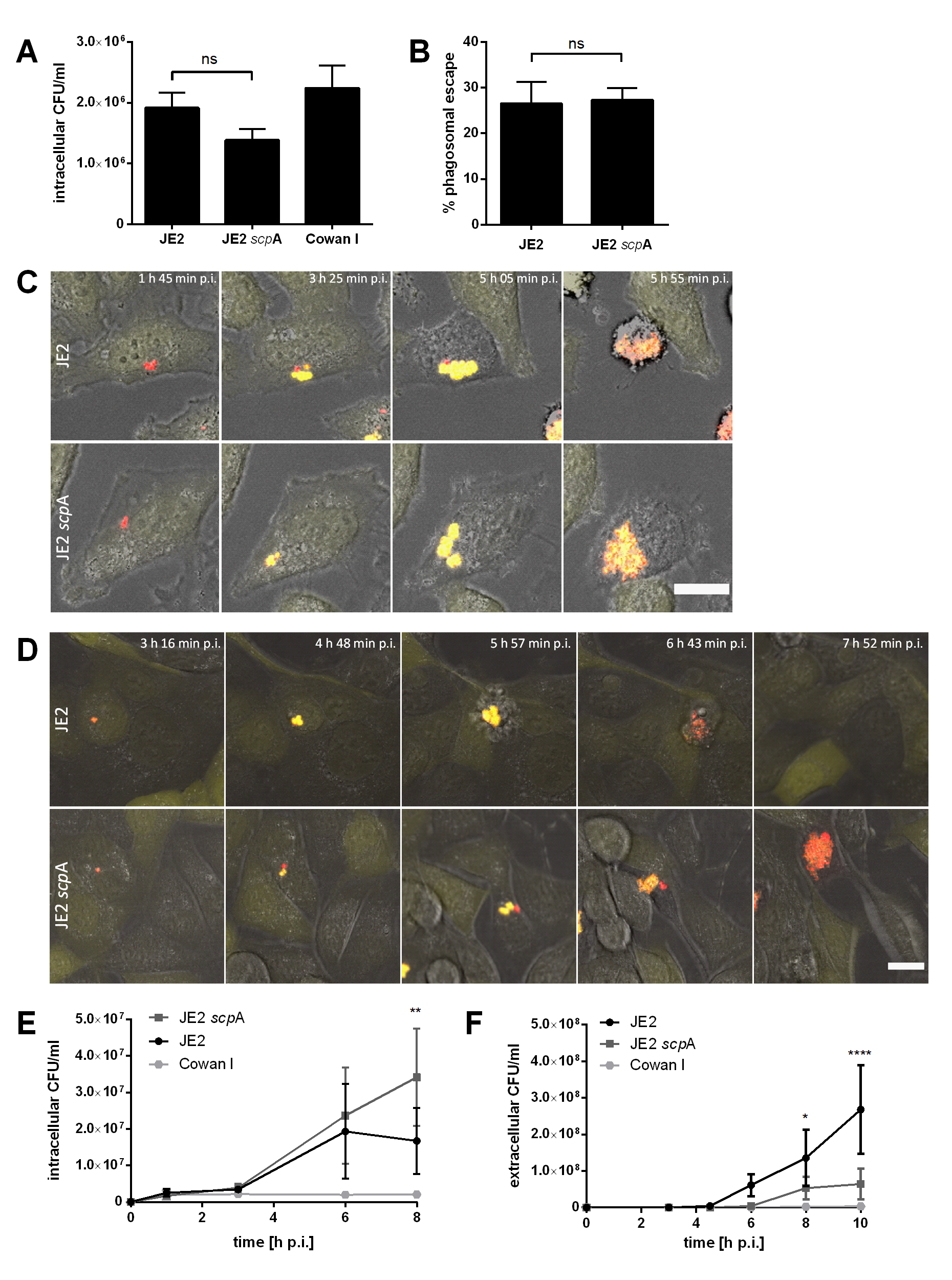

Supplement: S3 Fig — (A) HeLa cells were infected with JE2, JE2 scpA and Cowan I and invasion into HeLa cells was determined by quantifying intracellular CFUs at 1 h p.i. (B) Phagosomal escape was quantified in the marker cell line 16HBE14o- YFP-CWT 3 h p.i. by automated microscopy after infection with mRFP-expressing bacteria. (C) Infected HeLa YFP-CWT cells were imaged over time to visualize phagosomal escape of JE2 wild type (upper panel) and staphopain A mutant (JE2 scpA, lower panel) (red: S. aureus, yellow: YFP-CWT, gray: BF, scale bar: 20 μm). (D) Live cell imaging of 16HBE14o- YFP-CWT cells infected with S. aureus JE2 mRFP or JE2 scpA mRFP (scale: 20 μm). (E) HeLa cells were infected with JE2, JE2 scpA and Cowan I and intracellular bacterial CFUs were quantified at 1, 3, 6 and 8 h p.i. (F) HeLa cells were infected with JE2, JE2 scpA and Cowan I and only a lysostaphin-pulse was applied to remove extracellular bacteria after invasion. Bacteria, which escaped from the host cell, were quantified by CFU plating at 3, 4.5, 6, 8 and 10 h p.i. Statistical significance was determined by unpaired t-test (B), one-way ANOVA (A) or two-way ANOVA comparing JE2- to JE2 scpA-infected samples (E, F) (*P<0.05, **P<0.01, ****P<0.0001). (TIF) [file ppat.1009874.s003.tif]

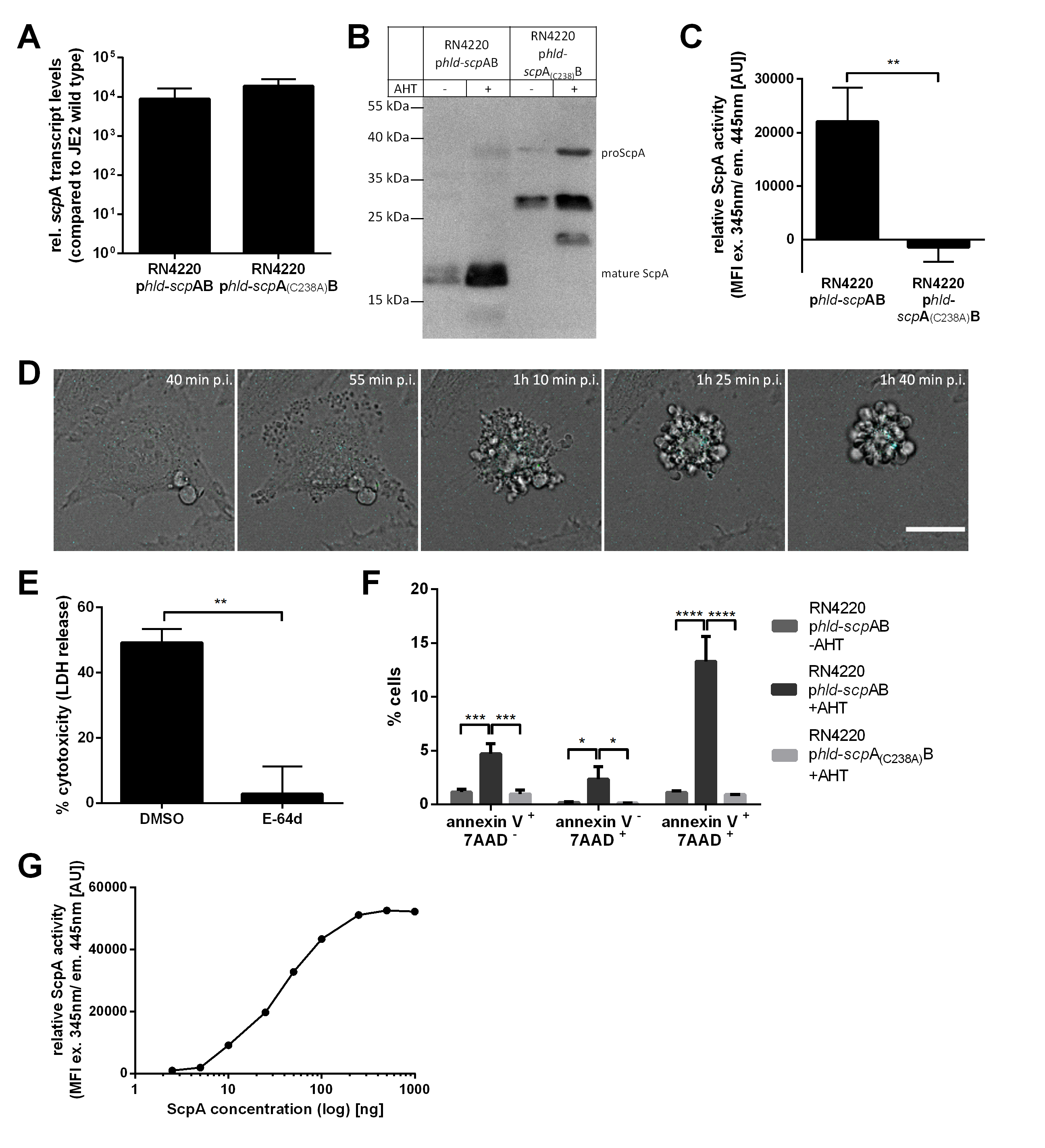

Supplement: S4 Fig — (A) RNA was isolated from bacterial overnight cultures and expression of scpA in RN4200 phld-scpAB and RN4220 phld-scpA(C238A)B was determined by qRT-PCR and normalized to scpA expression of JE2 wild type. (B) S. aureus RN4220 phld-scpAB or RN4220 phld-scpA(C238A)B were grown overnight with or without addition of 200 ng/ml AHT. Proteins of the sterile culture supernatant were precipitated and western blot was performed to detect staphopain A. Expression of functional ScpA was detected as the mature protein (triplet ranging from 17 to 20 kDa), while expression of a non-functional staphopain A led to accumulation of proScpA (ca. 40 kDa) [43]. (C) Proteolytic activity of staphopain A was measured from sterile culture supernatant of S. aureus RN4220 phld-scpAB or RN4220 phld-scpA(C238A)B. (D) Imaging of HeLa cells infected with S. aureus RN4220 phld-scpAB visualized cell contraction over time (cyan: S. aureus, gray: BF, scale bar: 20 μm). (E) The effect of E-64d (80 μM) treatment on cytotoxicity of RN4220 phld-scpAB infected HeLa cells was determined by quantification of LDH release 6 h p.i. and compared to solvent control (DMSO). (F) HeLa cells were infected with S. aureus RN4220 phld-scpAB or RN4220 phld-scpA(C238A)B with or without addition of 200 ng/ml AHT prior to infection. 4.5 h p.i. cells were stained with annexin V-APC and 7AAD and analyzed by flow cytometry. (G) Proteolytic activity of staphopain A was measured from increasing concentrations of the purified enzyme. Statistical significance was determined by unpaired t test (C, E) or two-way ANOVA (F) (*P<0.05, **P<0.01, ***P<0.001, ****P<0.0001). (TIF) [file ppat.1009874.s004.tif]

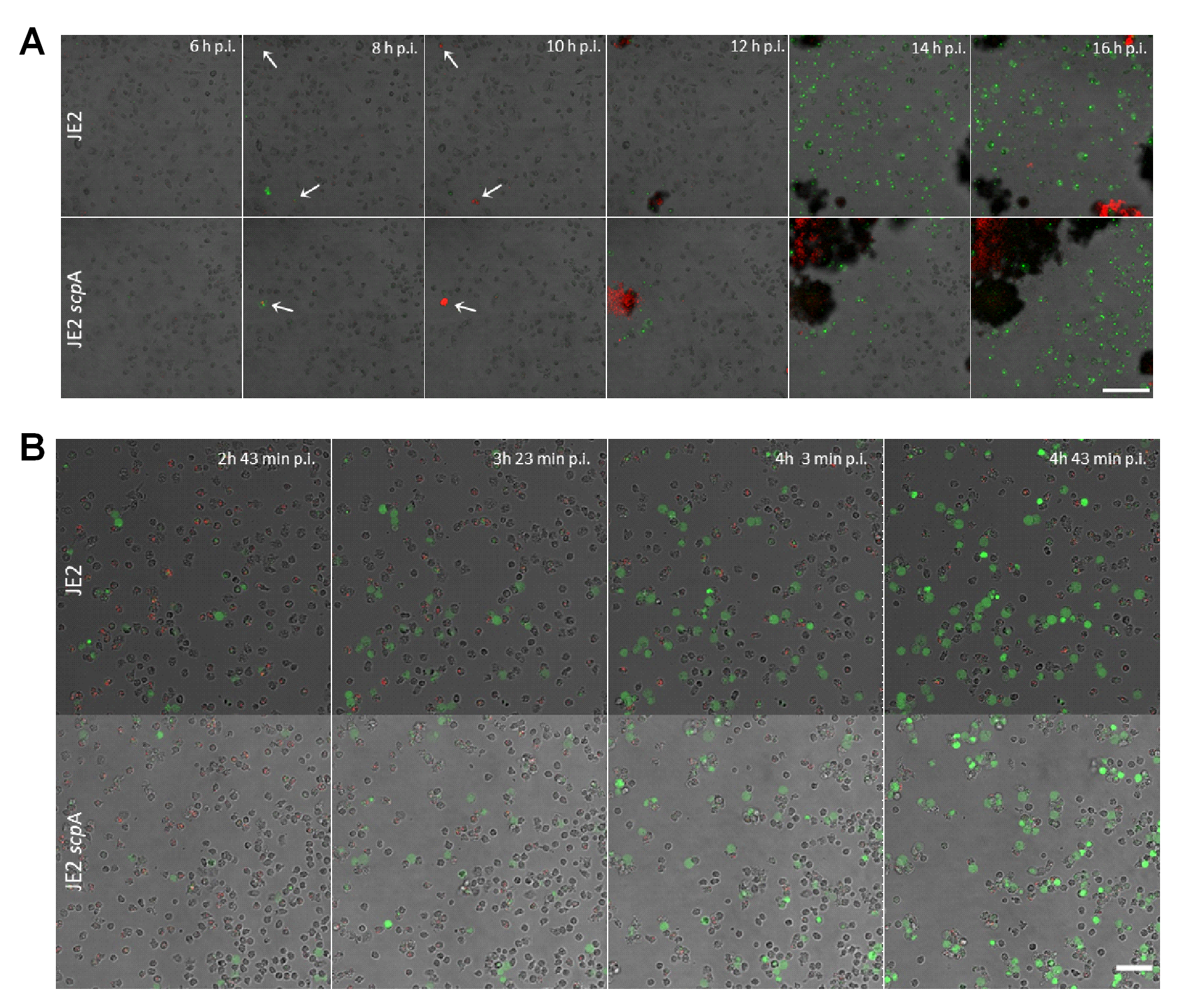

Supplement: S7 Fig — (A) Primary human M-CSF derived macrophages (A) or PMNs (B) were infected with S. aureus JE2 mRFP and JE2 scpA mRFP. Live cell imaging was performed to visualize infection (red: S. aureus, green: CellEvent Caspase3/7 Green Detection Reagent, gray: BF, scale bar: 200 μm (A)/50 μm (B)). (TIF) [file ppat.1009874.s007.tif]
