# Supplementary material for: Intracellular Staphylococcus aureus employs the cysteine protease staphopain A to induce host cell death in epithelial cells
Source: PLoS Pathog. 2021 Sep 2;17(9):e1009874. doi: 10.1371/journal.ppat.1009874 (PMC8443034; doi:10.1371/journal.ppat.1009874)
Supplement: S1 Table — (PDF) [file ppat.1009874.s012.pdf]

S1 Table. Bacterial strains used in this study.

Strain	Description	Source/Reference
Escherichia coli		
DH5α	*fhuA2 lacΔU169 phoA glnV44 Φ80' lacZΔM15 gyrA96 recA1 relA1 endA1 thi-1 hsdR17*	BRL Life Technology
Staphylococcus aureus		
RN4220	Restriction-deficient derivative of NCTC 8325-4 (cured of prophages Φ11, Φ12, Φ13), β-toxin producer, no production of α-toxin or δ-toxin, phenotypically *agr*-negative	[1]
RN4220 p*hld-scp*AB	RN4220 p*hld-scp*AB-cerulean, AHT-inducible expression of δ-toxin (*hld*), staphopain A (*scp*A), staphostatin A (*scp*B) and cerulean	This study
RN4220 p*hld-scp*$A_{(C238A)}$B	RN4220 p*hld-scp*$A_{(C238A)}$B-cerulean, AHT-inducible expression of δ-toxin (*hld*), staphopain A (*scp*A), staphostatin A (*scp*B) and cerulean with active site substitution C234A in *scp*A	This study
JE2	derivative of LAC, which was cured of three plasmids, USA300 PFGE type, CA-MRSA	[2]
JE2 GFP	JE2 pGFPsf, JE2 expressing GFPsf as molecular marker	This study
JE2 mRFP	JE2 pmRFPmars, JE2 expressing mRFP as molecular marker	This study
JE2 NE934	JE2 *sspB*::*bursa*, deficient in staphopain B production, SAUSA300_0950, ErmR	[2]
JE2 NE1278	JE2 *scp*A::*bursa*, deficient in staphopain A production, SAUSA300_1890, ErmR	[2]
JE2 *scp*A	JE2 *scp*A::*bursa*, staphopain A transposon mutant, produced by phage transduction from NE1278 in JE2	This study
JE2 *scp*A GFP	JE2 *scp*A::*bursa* pGFPsf, JE2 *scp*A expressing GFP as molecular marker	This study
JE2 *scp*A mRFP	JE2 *scp*A::*bursa* pmRFPmars, JE2 *scp*A expressing mRFP as molecular marker	This study
JE2 p*scp*AB	JE2 *scp*A::*bursa* p*scp*AB, *scp*A complementation in JE2 *scp*A	This study
JE2 p*scp*$A_{(C238A)}$B	JE2 *scp*A::*bursa* p*scp*$A_{(C238A)}$B, JE2 p*scp*AB with active site substitution C234A in *scp*A	This study

JE2 pP*scp*AB-GFP_P1*sar*A-mRFP	Promotor-reporter strain, GFP expression under *scp*AB promotor and mRFP expression under *sar*A P1 promoter	This study
6850	Clinical osteomyelitis isolate, methicillin-sensitive	[3]
6850 *scp*A	6850 *scp*A::*bursa*, staphopain A transposon mutant, produced by phage transduction from NE1278 in 6850	This study
6850 p*scp*AB	6850 *scp*A::*bursa* p*scp*AB, *scp*A complementation in 6850 *scp*A	This study
Cowan I	NCTC 8530, isolated from septic arthritis, *agr* dysfunction, low expression of toxins and proteases	ATCC 12598

References

1. Kreiswirth BN, Lofdahl S, Betley MJ, O'Reilly M, Schlievert PM, Bergdoll MS, et al. The toxic shock syndrome exotoxin structural gene is not detectably transmitted by a prophage. Nature. 1983;305(5936):709-12. Epub 1983/10/20. PubMed PMID: 6226876.
2. Fey PD, Endres JL, Yajjala VK, Widhelm TJ, Boissy RJ, Bose JL, et al. A genetic resource for rapid and comprehensive phenotype screening of nonessential Staphylococcus aureus genes. MBio. 2013;4(1):e00537-12. Epub 2013/02/14. doi: 10.1128/mBio.00537-12. PubMed PMID: 23404398; PubMed Central PMCID: PMCPMC3573662.
3. Vann JM, Proctor RA. Ingestion of Staphylococcus aureus by bovine endothelial cells results in time- and inoculum-dependent damage to endothelial cell monolayers. Infect Immun. 1987;55(9):2155-63. Epub 1987/09/01. PubMed PMID: 3623696; PubMed Central PMCID: PMCPMC260672.
