# Supplementary material for: Intracellular Staphylococcus aureus employs the cysteine protease staphopain A to induce host cell death in epithelial cells
Source: PLoS Pathog. 2021 Sep 2;17(9):e1009874. doi: 10.1371/journal.ppat.1009874 (PMC8443034; doi:10.1371/journal.ppat.1009874)
Supplement: S2 Table — (PDF) [file ppat.1009874.s013.pdf]

S2 Table. Plasmids used in this study.

Plasmid	Description	Reference
p2085	derivate of pALC2084 [1] with modified multiple-cloning site, shuttle vector, containing *tetR* and Pxyl/tet promoter driving GFPuvr expression, CmR	[2]
p*scp*AB	p2085-*scp*AB, complementation of *scp*A, native promotor of *scp*AB	This study
p*scp*$A_{(C238A)}$B	p*scp*AB with active site substitution C234A in *scp*A	This study
p*hld*-*hlb*-cerulean	p2085-*hld*-*hlb*-cerulean, AHT-inducible expression	[3]
p*hld*-*scp*AB-cerulean	p2085-*hld*-*scp*AB-cerulean, AHT-inducible expression	This study
p*hld*-*scp*$A_{(C238A)}$B-cerulean	p*hld*-*scp*AB-cerulean with active site substitution C234A in *scp*A	This study
pmRFPmars	p2085-SarAP1-mRFPmars, vector expressing mRFP in *S. aureus* under control of the constitutive sarAP1 promoter, CmR	[4]
pSK5632	*S. aureus*-*E. coli* shuttle vector, low copy plasmid, CmR	[5]
pGFPsf	pSK5632-SarAP1-GFPsf, vector expressing GFPsf in *S. aureus* under control of the constitutive sarAP1	This study
pP*scp*AB-GFP_P1*sar*A-mRFP	p2085-P*scp*AB-GFP_P1*sar*A-mRFP	This study

References

1. Bateman BT, Donegan NP, Jarry TM, Palma M, Cheung AL. Evaluation of a tetracycline-inducible promoter in Staphylococcus aureus in vitro and in vivo and its application in demonstrating the role of sigB in microcolony formation. Infect Immun. 2001;69(12):7851-7. Epub 2001/11/14. doi: 10.1128/IAI.69.12.7851-7857.2001. PubMed PMID: 11705967; PubMed Central PMCID: PMCPMC98881.
2. Giese B, Dittmann S, Paprotka K, Levin K, Weltrowski A, Biehler D, et al. Staphylococcal alpha-toxin is not sufficient to mediate escape from phagolysosomes in upper-airway epithelial cells. Infect Immun. 2009;77(9):3611-25. Epub 2009/07/01. doi: 10.1128/IAI.01478-08. PubMed PMID: 19564384; PubMed Central PMCID: PMCPMC2738027.
3. Giese B, Glowinski F, Paprotka K, Dittmann S, Steiner T, Sinha B, et al. Expression of delta-toxin by Staphylococcus aureus mediates escape from phago-endosomes of human epithelial and endothelial cells in the presence of beta-toxin. Cell Microbiol. 2011;13(2):316-29. Epub 2010/10/16. doi: 10.1111/j.1462-5822.2010.01538.x. PubMed PMID: 20946243.
4. Paprotka K, Giese B, Fraunholz MJ. Codon-improved fluorescent proteins in investigation of Staphylococcus aureus host pathogen interactions. J Microbiol Methods. 2010;83(1):82-6. Epub 2010/08/17. doi: 10.1016/j.mimet.2010.07.022. PubMed PMID: 20708040.

5. Grkovic S, Brown MH, Hardie KM, Firth N, Skurray RA. Stable low-copy-number Staphylococcus aureus shuttle vectors. Microbiology. 2003;149(Pt 3):785-94. Epub 2003/03/14. doi: 10.1099/mic.0.25951-0. PubMed PMID: 12634346.
