# Supplementary material for: Intracellular Staphylococcus aureus employs the cysteine protease staphopain A to induce host cell death in epithelial cells
Source: PLoS Pathog. 2021 Sep 2;17(9):e1009874. doi: 10.1371/journal.ppat.1009874 (PMC8443034; doi:10.1371/journal.ppat.1009874)
Supplement: S3 Table — (PDF) [file ppat.1009874.s014.pdf]

S3 Table. Oligonucleotides used in this study.

Name	Sequence (5´ to 3´)	Purpose
*scp*AB_prom_fwd_PstI	AAACTGCAGTATTCTATTGCATAGGTGTGG	Amplification of *scp*AB including promotor region with restriction sites for cloning of p*scp*AB
*scp*AB_TT_rev_EcoRI	AAGAATTCCTATTTGAAGAGGAAAGGCTATTC	
MP_*scp*A1	CTCAAGGTAACAATGGTTGGgcgGCAGGCTATACGATGTCT	Cys_{238}>Ala active site substitution in *scp*A
MP_*scp*A2	AGACATCGTATAGCCTGCcgcCCAACCATTGTTACCTTGAG	
sarAP-F-AflIII	ATATAACATGTTGCATGCCTGATATTTTTG	Amplification of SarAP1-mRFP from pmRFPmars with restriction sites for cloning of pGFPsf
Cer-R-KasI	AATTAGGCGCCAGAAACCTTGTTTTAC	
SfGFP-F-AvrII	AATTACCTAGGAGGTTTAAACATGTCA	Amplification of GFPsf with restriction sites for cloning of pGFPsf
SfGFP-R-BamHI	ATATAGGATCCCATGAGGCCCAGGAATTC	
*scp*AB_AvrII_fwd	GATCCCTAGGAGGTATAATAATGAAAAGAAACTTTCC	Amplification of *scp*AB with restriction sites for cloning of p*hld-scp*AB
*scp*AB_AvrII_rev	CATGGATCCTAGGTTATGACTTATGCTTAATGAAAG	
RT-*gyr*B-fwd	CGACTTTGATCTAGCGAAAG	qRT-PCR for *gyr*B
RT-*gyr*B-rev	ATAGCCTGCTTCAATTAACG	
RT-*scp*A-fwd	TTAATGTCGAGGACAAGAGTG	qRT-PCR for *scp*AB
RT-*scp*A-rev	GGTTCACCAAGTGTATACGAG	
*scp*AP+SphI_fwd	AAAGCATGCTATTCTATTGCATAGGTG	Amplification of *scp*AB promoter with restriction sites for SphI and PmeI
*scp*AP+PmeI_rev	AAAGTTTAAACATAAAAACTCCTTTATTTTATTATA	
