# Supplementary material for: Intracellular Staphylococcus aureus employs the cysteine protease staphopain A to induce host cell death in epithelial cells
Source: PLoS Pathog. 2021 Sep 2;17(9):e1009874. doi: 10.1371/journal.ppat.1009874 (PMC8443034; doi:10.1371/journal.ppat.1009874)
Supplement: Attachment — Submitted filename: Cover letter.docx [file ppat.1009874.s015.docx]

|  | **Theodor-Boveri Institut für Biowissenschaften**  **Lehrstuhl für Mikrobiologie** |  |
| --- | --- | --- |
|  |  |  |

| Lehrstuhl Mikrobiologie/Biozentrum, Am Hubland, 97074 Würzburg  To the Editor of  *PLOS pathogens*  Michael Otto | **Prof. Dr. Thomas Rudel** Sekretariat: J. Hümmer/K. Plaschek  Telefon 0931 / 318-4400  Telefax 0931 / 318-4402  Sekretariat.mikrobiologie@biozentrum.uni-wuerzburg.de  www.mikrobio.biozentrum.uni-wuerzburg.de  Würzburg, 03.08.2021 |
| --- | --- |

Submission of revised version of PPATHOGENS-D-21-01238

Dear Dr. Otto:

Thank you for considering our manuscript for publication in PLOS pathogens! We are submitting now a revised version of the previous manuscript PPATHOGENS-D-21-01238 entitled “*Intracellular Staphylococcus aureus employs the cysteine protease staphopain A to induce host cell death in epithelial cells*”*.*

Whereas reviewer 1 and 2 have only minor points, the new reviewer 3 suggested to perform further experiments to investigate the role of pyroptosis in staphopain A induced cell death. Since the form of cell death we characterized is mainly induced in epithelial cells and pyroptosis is a pro-inflammatory cell death pathway mostly observed in immune cells, we feel that the expected data of such experiments will not add significantly to the current manuscript. A detailed point-to-point response to the reviewer’s comments is included in this cover letter below.

We hope that our manuscript is now suitable for publication in PLOS pathogens and therefore are looking forward to your favorable response.

Sincerely,

Thomas Rudel

Reviewer's Responses to Questions

**Part I - Summary**
Please use this section to discuss strengths/weaknesses of study, novelty/significance, general execution and scholarship.

Reviewer #1:

This manuscript is a resubmission of previous work that describe a novel intracellular role of staphopain A in S. aureus pathogenesis.
Although the authors did not explore additional read outs in the mouse model of pneumonia as requested, the authors performed several new experiments that assessed the role of staphopain A in S. aureus pathogenesis in other cell types (this was greatly appreciated). An especially interesting result was that primary human macrophages and neutrophils were resistant to the effects of staphopain A cytotoxicity compared to epithelial cells. While the molecular mechanism for this specificity is unknown, identifying the intracellular target(s) as well as understanding how this relates to enhanced pathogenesis in vivo will be of particular interest.

Reviewer #2:

In this study, the authors interrogate the role of cysteine proteases in the intracellular lifestyle of S. aureus. They report that staphopain A plays a role in intracellular killing of the host cell after S. aureus escapes the phagosome. Inactivation of staphopain A in a cytotoxic S. aureus strain delayed onset of host cell death in epithelial cells whereas Inducible expression of the cysteine protease in a non-cytotoxic bacterial strain initiated apoptotic cell death. Finally, the authors report that staphylopain is important in a S. aureus lung colonization model. The manuscript is clear and well put together. The experiments are incisive, and the conclusions are supported by the data. I have a few minor suggestions or questions that can be addressed with text modifications.

Reviewer #3:

This paper describes the role of the cystein protease scpA in the cellular toxicity of S. aureus once it has been released in the cytosol from non professional phagocytes. The loss of this protein prolongs the survival of the bacteria in the cytosol of the cells, where they continue to multiply.
The experimental design is well constructed and the results are convincing, with appropriate controls included in each experiment.
The discoveries are original and significantly contribute to the understanding of the factors governing the ability of S. aureus to survive inside the cells or to escape from them.
The main weakness is that the authors did not manage to identify the type of cell death associated with the activity of this enzyme neither its substrate(s) in the cytosol. These two elements are probably closely related.

**Part II – Major Issues: Key Experiments Required for Acceptance**
Please use this section to detail the key new experiments or modifications of existing experiments that should be absolutely required to validate study conclusions.

Generally, there should be no more than 3 such required experiments or major modifications for a "Major Revision" recommendation. If more than 3 experiments are necessary to validate the study conclusions, then you are encouraged to recommend "Reject".

Reviewer #1: NA

Reviewer #2: None

Reviewer #3:

1. A key observation that is not exploited is the fact that cell toxicity is delayed in the absence of the cystein protease scpA but not reduced (figure 1). This observation should be better integrated in the interpretation of the mechanisms.

Authors’ Response:

The delayed cell death suggests that upon inactivation of Staphopain A another virulence factor initiates host cell death – although at later time points during infection. *S. aureus* expresses various virulence factors, of which to date only few have been shown to contribute to intracellular virulence.

We recently found that the pathogen can perturb the intracellular Ca^2+^-homeostasis of the host cell (1). This process occurs only after bacterial intracellular replication and is independent of ScpA.

We therefore believe that *S. aureus* not only employs staphopain A but also other factors to induce host cell death. These secondary virulence factor(s) may be not as efficient in initiating host cell death, whereas Staphopain A is causing this process rather efficiently and fast. Overexpression of *scp*A in a non-cytotoxic strain led to very rapid host cell death when the pathogen entered the cytoplasm (see Figure 5A). Hence, rather a delay than attenuation of cell death is observed in absence of ScpA.

We added this explanation and hypothesis to the discussion in the manuscript (see lines 495ff).

2. Another cell death mechanism compatible with Annexin V labeling and permeation of an intercalating agent is pyroptosis, which also involves caspase activation. This mechanism has not been explored here although it has been described for S. aureus.

Authors’ Response:

Pyroptosis is a pro-inflammatory cell death pathway, which is mostly observed in immune cells (2). We observed that overexpression of staphopain A by a non-cytotoxic *S. aureus* strain induces immediate host cell death with features of apoptosis and necrosis in HeLa cells. To our knowledge, contribution of pyroptosis to cell death of HeLa is negligible.

Induction of pyroptosis by *S. aureus* has been described so far mainly in immune cells and in keratinocytes (3). We also performed experiments with primary human macrophages and PMNs and did not see any effect of staphopain A in immune cells infected with cytotoxic *S. aureus* strains (see Figure 7B-F). When we used the non-cytotoxic *S. aureus* strain RN4220 to induce overexpression of *scp*A in human primary PMNs, as we did in HeLa cells, the bacteria were killed by neutrophils and no host cell death was visible (data not shown). Therefore, we conclude that pyroptosis, which is most relevant in immune cells, may not be the cell death pathway induced by staphopain A.

Nevertheless, we are interested in cytosolic host targets of ScpA in order to characterize the molecular mechanism of this protease within the host cell. We therefore currently design an experiment by which we want to identify, if specific targets of ScpA within the host cytoplasm exist or if the protease rather unspecifically degrades host components thereby killing the host cells. These experiments will be part of a future study and may well resolve the induction of a specific form of cell death by the bacteria.

3. In vivo data do not allow to distinguish between toxicity mediated by extracellular or intracellular bacteria and are thus difficult to reconcile with the role of scpA in intracellular survival.

Authors’ Response:

We agree with the reviewer. In our animal experiment we cannot exclude also extracellular effects of ScpA, as we point out in the discussion. If possible, animal experiments have to be designed in such a way that extra- and intracellular effects of staphopain A can be discriminated. To our knowledge, no such animal model has been developed yet, but will be required for a better understanding of intracellular *S. aureus* infection *in vivo*. This assessment has been added to the discussion (see lines 562-564).

**Part III – Minor Issues: Editorial and Data Presentation Modifications**
Please use this section for editorial suggestions as well as relatively minor modifications of existing data that would enhance clarity.

Reviewer #1: NA

Reviewer #2:

It is not clear why the authors used the murine pneumonia model to test the importance of staphopain in vivo, there are murine models that more accurately represent S. aureus replication. Perhaps some text could be added to the manuscript to justify this decision?

Authors’ Response:

We chose the murine pneumonia model, because we observed a strong phenotype of the *rsp* mutant in this animal model (4). Infection with the *rsp* mutant led to reduced lethality when compared to wild type- or complementation strain-infection. Staphopain A expression is dependent on Rsp and the *rsp* mutant shows a similar phenotype in epithelial cells, such as prolonged intracellular residence and reduced cytotoxicity, compared to mutation of *scp*A. Based on these observations, we were interested, if infection of the murine pneumonia model with our *scp*A mutant shows a similar effect. However, we were not able to investigate lethality in this model, as the animals suffer too much from this infection and we are legally not allowed to perform this experiment. Nevertheless, we observed reduced survival of the *scpA* mutant in murine pneumonia when compared to the wild type strain (see Figure 7A).

Is Rsp responsible for the altered expression of staphopain observed in Figure 4?

Authors’ Response:

Rsp previously has been shown to affect transcription of *scp*A (4). However, we currently do not know, if Rsp is responsible for the altered expression of staphopain A under these conditions. Future investigations will have to resolve the complex regulatory pattern of intracellular gene regulation with respect to the subcellular localization of the bacteria in their respective host cells.

Reviewer #3:

1. The way the data on immune cells are explained in the text is rather confusing, the discussion is much clearer in this respect.

Authors’ Response:

We rephrased the section on our findings in immune cells in the results part to make it more comprehensible for the reader (see lines 387ff).

2. How were normalized bacterial counts ? The scale is in CFU/ml (fig S3 E, for example), but per ml of what ? the number of cells is decreasing during the experiment.

Authors’ Response:

We quantified CFU per well, as we cultivated the infected cells in 1 ml infection medium and for CFU counts lyse the cells in 1 ml alkaline water. We add the same number of cells and bacteria to each well. Therefore, CFU/ml means CFU/well.

Infected cells are killed by intracellular *S. aureus* over the time course of infection and extracellular bacteria emerging from dying cells are killed by the antibiotic added to the medium. We are aware that numbers of host cells and bacteria are thus decreasing over time. This is also obvious in our experiments (see Figure 3D and S3E). Bacterial counts of the wild type drop earlier compared to the *scp*A mutant due to the delayed cytotoxicity of the mutant.

By contrast, bacteria released by dead host cells were able to grow in the culture media when we only apply an initial antibiotic pulse and omitted lysostaphin from the medium in the remainder of the time course (see Figure S3F). Here, outgrowth of wild type bacteria commences earlier when compared to the *scp*A mutant, because inactivation of *scp*A leads to a delay of intracellular *S. aureus* cytotoxicity.

To make this clearer to the readers, we added an explanation in the methods section of the manuscript (see lines 828ff).

3. Any idea about the factor(s) that could activate the expression of scpA in the cytosol ? Is this a question of pH ?

Authors’ Response:

ScpA expression is significantly influenced by SarA (repressor) and SarR (activator) (5), but gene transcription is also influenced by other transcription factors such as Rsp (4).

Our unpublished data show that cellular H_2_O_2_ concentration is elevated during intracellular *S. aureus* infection. We observe this after phagosomal escape of the pathogen. Therefore, we could hypothesize that this rise in H_2_O_2_ leads to increased *scp*A expression mediated by rsp. Treatment of *S. aureus* with H_2_O_2_ led to increased *rsp* expression, which is in turn enhancing *scp*A expression (4). Thus, oxidative stress may account for that induction pattern.

4. Figure 5. What is the concentration of scpA that can be found intracellularly ? Is there any evidence that it is in the range of concentrations of purified protein investigated here ?

Authors’ Response:

We currently do not know the concentration of ScpA protein in infected host cells or host tissue. To our knowledge, this was not investigated so far. We therefore chose the tested concentrations of purified staphopain A based on the results from the activity assay (see Figure S4G).

ScpA antibody was not showing clear results in Western Blots. Thus it was not possible to estimate protein abundance by this method. However, if staphopain A would induce a proteolytic cascade leading up to cell death, only small amounts of active protease may be required to initiate host cell death.

RNA expression by RT-PCR and fluorescence reporter (see Figure 4) however, show -if only indirectly- activity of the promoter and transcription of the gene.

References:

1.	Stelzner, K., Winkler, A.C., Liang, C., Boyny, A., Ade, C.P., Dandekar, T., Fraunholz, M.J. and Rudel, T. (2020) Intracellular Staphylococcus aureus Perturbs the Host Cell Ca(2+) Homeostasis To Promote Cell Death. *mBio*, **11**.

2.	Yu, P., Zhang, X., Liu, N., Tang, L., Peng, C. and Chen, X. (2021) Pyroptosis: mechanisms and diseases. *Signal Transduct Target Ther*, **6**, 128.

3.	Soe, Y.M., Bedoui, S., Stinear, T.P. and Hachani, A. (2021) Intracellular Staphylococcus aureus and host cell death pathways. *Cell Microbiol*, **23**, e13317.

4.	Das, S., Lindemann, C., Young, B.C., Muller, J., Osterreich, B., Ternette, N., Winkler, A.C., Paprotka, K., Reinhardt, R., Forstner, K.U. *et al.* (2016) Natural mutations in a Staphylococcus aureus virulence regulator attenuate cytotoxicity but permit bacteremia and abscess formation. *Proc Natl Acad Sci U S A*, **113**, E3101-3110.

5.	Gimza, B.D., Larias, M.I., Budny, B.G. and Shaw, L.N. (2019) Mapping the Global Network of Extracellular Protease Regulation in Staphylococcus aureus. *mSphere*, **4**.
